# Neutrophil Lymphocyte Ratio as a predictor of systemic inflammation - A cross-sectional study in a pre-admission setting.

**DOI:** 10.12688/f1000research.6474.1

**Published:** 2015-05-22

**Authors:** Lashmi Venkatraghavan, Tze Ping Tan, Jigesh Mehta, Anil Arekapudi, Arun Govindarajulu, Eric Siu

**Affiliations:** 1 Department of Anesthesia, Toronto Western Hospital, University Health Network, Toronto, Ontario, M5T 2S8, Canada

**Keywords:** Neutrophil Lymphocyte Ratio, Revised Cardiac Risk Index

## Abstract

*Background:* Neutrophil:lymphocyte ratio (NLR)  is an emerging biomarker that is used to predict postoperative mortality and morbidity in cardiac and cancer surgeries. The association of this biomarker with systemic illness and its usefulness in risk assessment of preoperative patients has not been fully elucidated.

*Objectives:* To determine the prevalence of elevated NLR in preoperative patients and to examine the relationship between elevated NLR and the presence of systemic illnesses as well as anaesthesia risk indices such as American Society of Anesthesia (ASA) and the revised cardiac risk index (RCRI) scores.

*Design:* Cross-sectional study

*Setting:* Anaesthesia pre-admission clinic, Toronto Western Hospital, Toronto, Canada

*Patients:* We evaluated 1117 pre-operative patients seen at an anesthesia preadmission clinic.

*Results:* NLR was elevated (>3.3) in 26.6% of target population. In multivariate analysis, congestive cardiac failure, diabetes mellitus and malignancy were independent risk factors predicting raised NLR. After regression analysis, a relationship between NLR and ASA score (Odds Ratio 1.78; 95% CI: 1.42-2.24) and revised cardiac risk index (RCRI, odds ratio 1.33; 95% CI: 1.09-1.64, p-value: 0.0063) was observed.

*Conclusions: * NLR was elevated (> 3.3) in 26.6% of patients. Congestive cardiac failure and malignancy were two constant predictors of elevated NLR at >3.3 and > 4.5. There was a strong association between NLR and anesthesia risk scoring tools of ASA and RCRI.

## Introduction

Perioperative major cardiovascular adverse events contribute to significant morbidity and mortality in patients undergoing non-cardiac surgery ^1^. Prospective identification of high risk patients coming for surgery will allow hospitals to prioritise the allocation of valuable resources such as intensive care beds and to avoid unplanned readmissions ^2^. The use of the American Society of Anaesthesia (ASA) physical status as the sole risk stratification score may not give the complete picture of the patient’s underlying inflammatory processes and may be inadequate for identifying risk during the immediate postoperative period. Hence, in addition to risk indices, biomarkers may have a role in assisting risk prediction and aid in developing a management plan. Biomarkers such as B-natriuretic peptide and C-reactive peptide have been effectively used in the past to stratify risk and optimise patient care to minimise the adverse events in the perioperative setting ^3– 6^. However, these markers have some limitations in their role in routine clinical practice, as they have been shown to provide either modest or no improvement over conventional risk factors in predicting cardiovascular outcome in non-heart failure populations ^3^.

An ideal biomarker would be something that can be measured or derived from routine blood work done during the preoperative preparation of the patient for the surgery. It should also have a high predictive value for the risk factors of interest. The role of white cell counts in predicting cardiac morbidity and mortality after surgery is well recognised ^7– 9^. An elevated neutrophil count has been shown to correlate with worse outcomes in patients with acute coronary events ^10^.

Neutrophil:lymphocyte ratio (NLR), a ratio of the neutrophil to lymphocyte count, has recently been shown to be predictive of morbidity and mortality in patients with acute coronary syndrome ^11, 12^. In addition, elevated NLR is associated with increased in-hospital and post-hospital mortality as well as increased risk in myocardial infarction patients ^13– 15^. However, the role of NLR in the risk assessment of preoperative patients in the general surgical population has not been well studied, as prior studies have concentrated on specific cardiac, vascular and oncological diseases.

Therefore, we sought to study the normal values and distribution of preoperative NLR in patients before their surgery, with the primary objective of examining the prevalence of elevated NLR in the non-cardiac surgical population. Secondary objectives included examining the relationship between this biomarker and the presence of systemic illnessess and the anaesthesia risk indices such as the ASA physical status and the RCRI scores ^16^.

## Methods

Ethical approval for this study was provided by the University Health Network Research Ethics Board, Toronto, Canada on 26 ^th^ May 2013 (Protocol#: 13-6395; REB Co-chair: Anna Gagliard). Informed consent was waived by the REB for this study. This study complies with STrengthening the Reporting of Observational studies in Epidemiology (STROBE) statements for reporting of observational trials ^17^.

This cross-sectional study investigated all consecutive elective surgical patients who attended the anaesthesia pre-admission clinic at our institution from 1 ^st^ January 2013 to 30 ^th^ April 2013. The pre-admission clinic conducts preoperative preparation and anaesthetic evaluation of patients undergoing major orthopedic, neuro-, spine, bariatric and general surgeries. Orthopedic surgeries included knee or hip joint replacements, hand and foot surgeries; neurosurgery included craniotomies for tumors, clipping of aneurysms and transphenoidal pituitary surgeries; spinal surgeries included lumbar and cervical decompression and fusions and tumor resections, and general surgeries included bowel resections, hernia repairs and Roux en Y gastric bypasses.

All patients in this cohort who were eligible for a complete blood count test were included in this study. As per the hospital policy, all patients who were above 60 years of age or suspected of having anemia, or if they required a cross-matching for the surgical procedure, will undertake a complete blood count test. Patients who are not required to have this blood test preoperatively and those with incomplete blood test results were excluded from the study.

As per our clinic’s practice, all major surgical patients are assessed by a nurse, and clinical history and examinations are entered in an electronic ‘Clinical Anaesthesia Information System’ (CAIS). The data were identified retrospectively from the CAIS database and extracted for patients demographics (age, sex), anaesthetic variables (ASA status), surgical variables (type of surgery) and presence of systemic illness medications (number of medications, ischemic heart disease, congestive cardiac failure, hypertension, diabetes, chronic kidney disease, cerebrovascular disease, and oncological disease). RCRI was computed based on the variables obtained from the clinical database. The differential white blood cell count, containing the neutrophil and lymphocyte absolute counts, and platelet counts were retrieved separately from the hematology records in the hospital’s electronic patient record (EPR). NLR was calculated from the complete blood counts.

### Statistical analysis

Continuous variables were expressed as mean +/- standard deviation (SD). Categorical data were summarised using absolute values (percentage). Previous studies have shown that an NLR value of > 3.3 and > 4.5 was shown to predict increased risk of myocardial infarction in post-surgical and asymptomatic populations, respectively ^12, 13^. Hence we selected both cut-off values for dichotomous comparisons to categorise patients into normal or elevated NLR.

Welch’s t-test was used for comparison between groups for continuous variables. Effects of the different variables on NLR were calculated in univariate analysis. The candidate predictors for the multivariate model were selected by screening with univariate analysis with a threshold for inclusion of p < 0.25. Multiple logistic regressions were used to determine independent predictors for elevated NLR. A p-value of < 0.05 was considered statistically significant. All statistical analyses were performed using **R** (Version 3.02, The Comprehensive R Archive Network; http://cran.r-project.org, Accessed 17 ^th^ November 2014).

## Results

### Data sampling

One thousand, one hundred and seventy-three patients were identified through the CAIS database. Fifty-six patients were excluded from the study as there was either no complete blood count performed or the white cell count subtypes were not performed. Therefore, 1117 patients were included for further analysis.

The patient population had a mean (±SD) age of 59 (±14.9) years and was comprised of 54.3% females. The majority of patients had an ASA score of 2 or 3 (91.6%). Patients for orthopedic surgery were the largest group seen in the pre-admission clinic (44.0%), followed by neurosurgery (21.5%), spinal surgery (15.4%), bariatric surgery (7.9%) and the rest (11.2%) were for urology and general surgery.

Baseline characteristics of patients grouped according to NLR > 3.3 and NLR > 4.5 are summarised in. The median value of NLR in the study population was 2.20. About one fourth (26.6%) of patients had a NLR value > 3.3 and 9.04% patients had a NLR value > 4.5. In univariate analysis using NLR > 3.3 as a threshold, age, congestive cardiac failure, chronic kidney disease, malignancy and the number of prescribed medications were increasingly associated. Congestive cardiac failure, diabetes mellitus and malignancy were the independent risk factors associated with elevated NLR after multivariate analysis ( Table 2).

**Table 1.** Characteristics of patients with NLR thresholds > 3.3 and > 4.5.

Characteristic	<=3.3	>3.3	<=4.5	>4.5
**N**	882(79)	235(21)	1016(90.9)	101(9.1)
**Mean Age (SD)**	58 (14.63)	62 (15.44)	59 (14.79)	63 (15.82)
**Female**	487(55.2)	119(50.6)	561(55.2)	45(44.6)
**ASA1**	43(4.9)	6(2.6)	47(4.6)	2(2.0)
**2**	411(46.6)	77(32.8)	464(45.7)	24(23.8)
**3**	405(45.9)	141(60.0)	478(47.0)	68(67.3)
**4**	23(2.6)	11(4.7)	27(2.7)	7(6.9)
**5**	0(0.0)	0(0.0)	0(0.0)	0(0.0)
**IHD**	98(11.1)	33(14.0)	115(11.3)	16(15.8)
**CHF**	28(3.2)	21(8.9)	36(3.5)	13(12.9)
**HT**	404(45.8)	119(50.6)	474(46.7)	49(48.5)
**Diabetes**	141(16.0)	41(17.4)	162(15.9)	20(19.8)
**CV**	37(4.2)	12(5.1)	46(4.5)	3(3.0)
**Malignancy**	87(9.9)	40(17.0)	109(10.7)	18(17.8)
**CKD Stage:1**	423(48.3)	71(30.3)	466(46.2)	28(28.0)
**2**	207(23.7)	62(26.5)	244(24.2)	25(25.0)
**3**	80(9.1)	43(18.4)	102(10.1)	21(21.0)
**4**	6(0.7)	3(1.3)	8(0.8)	1(1.0)
**5**	6(0.7)	4(1.7)	8(0.8)	2(2.0)
**Medications :0**	252(28.6)	47(20.0)	279(27.5)	20(19.8)
**1**	308(34.9)	82(34.9)	362(35.6)	28(27.7)
**2**	167(18.9)	52(22.1)	195(19.2)	24(23.8)
**3**	106(12)	33(14)	123(12.1)	16(15.8)
**4**	36(4.1)	16(6.8)	43(4.2)	9(8.9)
**5**	10(1.1)	4(1.7)	11(1.1)	3(3)
**6**	2(0.2)	1(0.4)	2(0.2)	1(1)
**RCRI :0**	634(71.9)	147(62.6)	718(70.7)	63(62.4)
**1**	193(21.9)	66(28.1)	232(22.8)	27(26.7)
**2**	43(4.9)	11(4.7)	50(4.9)	4(4.0)
**3**	10(1.1)	7(3.0)	12(1.2)	5(5.0)
**4**	2(0.2)	3(1.3)	3(0.3)	2(2.0)
**5**	0(0.0)	1(0.4)	1(0.1)	0(0.0)

**Data layout:** Numbers of patients (percentage of the total cohort). **Key:** ASA - American Society of Anaesthesiologist physical status score; IHD - Ischemic heart disease; CHF - Congestive heart failure; HT - Hypertension; CV - Cerebral Vascular; CKD stage 1–5 - Chronic kidney disease stage 1–5; RCRI - Revised Cardiac Risk Index Score.

**Table 2.** Results of univariate and multivariate logistic regression models for neutrophil lymphocyte ratio (NLR > 3.3).

Logistic regression	Univariate		Multivariate	
Variable	Unadjusted OR (95% CI)	p-value	Adjusted OR (95% CI)	p-value
**Age**	**1.02 (1.01–1.03)**	**<0.001**	**1.02 (1.01–1.03)**	**0.004**
**Gender**	1.20 (0.90–1.60)	0.211	1.16 (0.86–1.57)	0.343
**IHD**	1.31 (0.85–1.99)	0.218	0.95 (0.60–1.52)	0.836
**CHF**	**2.99 (1.66–5.37)**	**0.0002**	**2.28 (1.21–4.31)**	**0.011**
**HT**	1.21 (0.91–1.62)	0.187	0.87 (0.60–1.25)	0.439
**Diabetes**	1.11 (0.76–1.63)	0.59	0.83 (0.52–1.32)	0.43
**CV disease**	1.23 (0.63–2.40)	0.545	0.94 (0.46–1.89)	0.855
**CKD**	**1.25 (1.09–1.45)**	**0.002**	1.05 (0.89–1.23)	0.599
**Malignancy**	**1.90 (1.37–2.64)**	**0.0001**	**1.79 (1.28–2.50)**	**0.001**
**Number of Medications**	**1.18 (1.06–1.32)**	**0.003**	1.13 (0.96–1.33)	0.134

**Key**: OR - odds ratio; CI - confidence interval; IHD - Ischemic heart disease; CHF - Congestive heart failure; HT - Hypertension; CV - Cerebral Vascular; CKD - Chronic kidney disease. **Significant results in bold face.**

However, when NLR > 4.5 were analyzed, polypharmacy emerged as a risk factor along with congestive cardiac failure and malignancy ( Table 3). Diabetes mellitus was not an independent risk factor.

**Table 3.** Results of univariate and multivariate logistic regression models for neutrophil lymphocyte ratio (NLR > 4.5).

Logistic regression	Univariate		Multivariate	
Variables	Unadjusted OR (95% CI)	p-value	Adjusted OR (95% CI)	p-value
**Age**	**1.02 (1.00–1.03)**	**0.03**	1.01 (0.99–1.03)	0.20
**Gender**	**1.53 (1.02–2.32)**	**0.04**	1.42 (0.92–2.20)	0.11
**IHD**	1.47 (0.83–2.60)	0.18	0.89 (0.47–1.70)	0.72
**CHF**	**4.02 (2.05–7.86)**	**<0.01**	**2.86 (1.34– 6.10)**	**<0.01**
**HT**	1.08 (0.72–1.62)	0.72	0.62 (0.36–1.06)	0.08
**Diabetes**	1.30 (0.78–2.18)	0.31	0.73 (0.37–1.42)	0.35
**CV disease**	0.65 (0.20–2.11)	0.47	0.41 (0.12 –1.43)	0.16
**CKD stage**	**1.25 (1.03–1.52)**	**0.03**	1.03 (0.81–1.30)	0.83
**Malignancy**	**2.41 (1.63–3.56)**	**<0.01**	**2.26 (1.50–3.41)**	**<0.01**
**Number. of medications**	**1.30 (1.12–1.51)**	**0.0006**	**1.40 (1.11–1.76)**	**<0.01**

**Key:** OR - odds ratio; CI - confidence interval; IHD - Ischemic heart disease; CHF - Congestive heart failure; HT - Hypertension; CV - Cerebral Vascular; CKD - Chronic kidney disease. **Significant results in bold face.**

In addition, NLR was significantly elevated in patients with any type of malignancy compared to patients with no malignancy (mean NLR 3.42 vs 2.70, p = 0.049). Regression analysis showed a strong association between NLR and ASA scores (Odds Ratio 1.78; 95% CI: 1.42-2.24, p-value: <0.001) and also between NLR and RCRI (Odds Ratio 1.33; 95% CI: 1.09-1.64, p-value: 0.0063).

Neutrophil-lymphocyte ratio study - patient characteristics, co-morbidities, revised cardiac risk index, ASA status and NLR For Gender - 1: male, 0 - female; Type of Surgery: 1: Orthopedic, 2: Neurosurgery, 3: Spinal, 4: Bariatric, 5: Gen Surg/Colorectal, 6: Urological, 7: Others; BMI: 0: < 30, 1: 30–39.9, 2: > 40; For Comorbid conditions: 1: yes, have disease, 2: no disease; For smoking status: 0: Never smoked, 1: Previous or current smoker; For Chronic Kidney Disease Stage: 1 = eGFR > 90, 2 = 60–89, 3 = 30–59, 4 = 15–29, 5 = < 15 or in renal therapy;
For all medications (except HT meds): 1 = yes, takes medications, 2 = no medications; For HT meds: the number of medications; For RCRI - Revised Cardiac Risk Index ^29^

## Discussion

To our knowledge this is the first cross-sectional study on the prevalence of elevated NLR in a preoperative setting. In our patient population, 26.6% of patients had an elevated NLR value of more than 3.3, which had been shown to be associated with increased mortality after cardiac surgery ^18^. We found an independent association between congestive cardiac failure and NLR values > 3.3 and > 4.5. This is in line with established evidence on the utility of NLR in predicting long-term outcome in acutely decompensated heart failure ^19^. Our study also showed a strong association between the presence of malignancy and NLR thresholds of > 3.3 and > 4.5. This confirms the previously known prognostic value of NLR in terms of mortality and recurrence of disease in patients with cancer ^20^.

In addition, we found a significant association between NLR and ASA physical status and RCRI. This raised the possibility that NLR may be of added value in anaesthetic risk stratification and comparison in a preoperative setting. This may be particularly relevant in a tertiary hospital setting where patients often present with many comorbidities.

Our study also showed that increased use of medication is a risk factor associated with NLR values > 4.5. The increased number of medications reflects the presence of more systemic illnesses. The relationship between polypharmacy and comorbidity is a well-recognised phenomenon especially in the elderly ^21^. This also highlights the relationship between NLR and presence of increased disease burden in patients with chronic systemic illnesses.

However, we did not demonstrate an association between the presence of ischemic heart disease and elevated NLR, contrary to previous publications ^12^. It is not clear from our cohort the reason for this discrepancy. It may be possible that the inclusion criteria for ischemic heart disease in our database includes all patients from angina-like symptoms to patients with previous myocardial infarction, thus weakening the association between NLR and ischemic heart disease. Further, it is likely that the majority of patients who presented to the pre-surgical clinic had stable ischemic heart disease and are on statin therapy and therefore have suppression of low-grade inflammation, leading to a lower NLR than anticipated.

Prediction of systemic illness and its severity are crucial in formulating a safe intraoperative plan and postoperative care in surgical patients. Elevated NLR is known to predict all-cause mortality in cardiac and major vascular surgeries ^18, 22^. Elevated preoperative NLR is also associated with increased morbidity postoperatively, with prolonged hospitalization and increased intensive care admission ^18^. Further, elevated NLR is associated with increased morbidity and mortality in sepsis ^23^. Hence, the use of NLR as a potential biomarker for preoperative risk assessment and stratification can be pertinent for several reasons. Firstly, NLR is obtained from a complete blood count that is routinely performed pre and postoperatively; it doesn’t necessitate additional investigation and is an inexpensive and readily available marker. Secondly, the application of elevated NLR can be used to plan for the postoperative support and rehabilitation of patients undergoing major surgeries. Another similar finding has been observed in the general population cohort in the recently published post-hoc analysis of the National Health and Nutrition Examination Survey-III that showed that NLR can independently predict cardiac mortality. In analysis of 7363 subjects, Shah *et al.* showed that NLR can be used as a risk index to reclassify patients from low to intermediate risk categories and improve the Framingham risk score ^24^.

Elevated NLR has been shown to be associated with increased tumor necrosis factor (TNF) alpha, and various interleukins (IL-6, IL-7, IL-8, IL-12, IL-17) ^25, 26^. These markers are known to be associated with poor outcome in critically ill patients, as well as with increased incidence of recurrent ischemic events in cardiac patients ^27, 28^. However, measuring these biomarkers is expensive and tests are not routinely performed. In contrast, NLR is a simple index derived from routine blood tests which might provide equal and valuable information in the preoperative setting.

Our study suffers from some major limitations. This study is not designed to examine the utility of this biomarker for stratifying risk and predicting clinical end-points of mortality and major cardiovascular adverse events. Rather it is designed to provide a cross-sectional “snapshot” of the distribution of NLR in a cohort of patients who attended the anaesthesia pre-admission clinic before their surgery and to correlate this biomarker with the presence of systemic illnesses.

Further prospective cohort studies are needed to examine the optimal role of NLR as a biomarker in the perioperative setting. Establishing a relationship between the NLR and systemic illnesses in elective preoperative patients sets the stage for evaluating the role of this biomarker in future prospective studies looking at the ideal cut-off value of these biomarkers and the correlation between elevated NLR, and perioperative morbidities and mortalities. Prediction of systemic illness and its severity are crucial in formulating a safe intraoperative plan and postoperative care in surgical patients.

## Conclusion

In summary, elevated preoperative NLR (> 3.3) was observed in 26.6% of patients attending a pre-surgical anaesthetic clinic at our tertiary hospital. Congestive cardiac failure and malignancy were two constant associations of elevated NLR at > 3.3 and > 4.5 respectively. There was also a strong association between NLR, and ASA and RCRI scores of patients. More studies are needed to determine the utility of NLR as a biomarker in the preoperative risk stratification of patients.

## Data availability

*F1000Research*: Dataset 1. Neutrophil-lymphocyte ratio study - patient characteristics, co-morbidities, revised cardiac risk index, ASA status and NLR., 10.5256/f1000research.6474.d47345 ^29^
