# Supplementary material for: Neutrophil Lymphocyte Ratio as a predictor of systemic inflammation - A cross-sectional study in a pre-admission setting
Source: F1000Res. 2015 May 22;4:123. [Version 1] doi: 10.12688/f1000research.6474.1 (PMC4505778; doi:10.12688/f1000research.6474.1)
Supplement: Peer review history

# Peer review: Referee response for version 1

**DOI:** 10.5256/f1000research.6946.r9421

**Published:** 2015-07-13

**Authors:** Edwin Seet

**Affiliations:** 1 Department of Anaesthesia, Khoo Teck Puat Hospital, Yishun, Singapore

Dr Venkatraghavan and colleagues have undertaken a cross-sectional observation study in a tertiary centre in Toronto, evaluating the utility of a raised Neutrophil:Lymphocyte Ratio as a biomarker for risk stratification in the perioperative period. A raised NLR was compared with conventionally accepted risk assessment tools such as the ASA physical status and the RCRI scores. Multivariate regression analysis was done to investigate independent predictors of a raised NLR. The authors found that a raised NLR was associated with malignancy, congestive heart failure and polypharmacy. A relationship between raised NLR and 2 cutoffs and increased ASA physical status and RCRI scores was observed.

Several possible opportunities of improvement of the manuscript may exist:

1. This is a novel study investigating raised NLR and perioperative comorbidities and conventional risk stratification tools. Most readers would not be familiar with the biological plausibility of a raised NLR and perioperative outcomes. More details may be included in the introduction and discussion to make this clearer to the readers.
2. Dr Peter Nagele and colleagues published a landmark paper correlating a raised High-Sensitivity Troponin T test and perioperative outcomes in Nagele *et al* (2013). This publication should be referenced together with other biomarkers in the introduction and discussion such as the BNP and CRP.
3. The authors have chosen to represent the association between NLR and ASA physical status and RCRI scores in the form of Odds Ratio. I wonder if correlation statistics and/or Receiver Operating Characteristics at various cutoffs may be utilized and represented in the results as well?

All in all, Dr Venkatraghavan *et al* have undertaken an interesting study which answers a novel and fairly important clinical question. I would like to congratulate them on a stellar effort.

I have read this submission. I believe that I have an appropriate level of expertise to confirm that it is of an acceptable scientific standard, however I have significant reservations, as outlined above.

# Peer review: Referee response for version 1

**DOI:** 10.5256/f1000research.6946.r8736

**Published:** 2015-06-03

**Authors:** Neeraj N. Shah

**Affiliations:** 1 Department of Cardiology, Lehigh Valley Health Network, Allentown, PA, USA

Venkatraghavan et al present an interesting study of preoperative neutrophil to lymphocyte ratio (NLR) in patients undergoing non-cardiac surgeries. While NLR has been studied extensively in acute and chronic cardiac conditions and in the setting of cardiac surgeries, there is scarce data about NLR in non-cardiac surgeries. It is unfortunate that the authors did not look at mortality or morbidity endpoints, rather this is a cross sectional study looking at prevalence of risk factors and correlation with ASA and RCRI scores preoperatively.

Suggested revisions:

1. Introduction, first paragraph: In the sentence "Prospective identification of high risk pts..... and to avoid unplanned readmissions". How will identification of high risk preop patients avoid unplanned readmissions? This sentence is confusing and I would recommend the authors deleting the part which says "to avoid unplanned readmissions".

2. Results: Use numbers instead of words e.g. use 1,173 instead of one thousand one hundred seventy three.

3. Table 1: Please include type of surgery in table 1 (e.g. orthopedic, neurosurgery, etc.)

3. Table 1: Medications: Please lump together 4,5, or 6 medications as 4 or more medications, since the definition of polypharmacy is 4 or more medications.

4. (Major concern) Results: Table 2 clearly shows that for NLR > 3.3, the independent predictors are age, CHF and malignancy, but the authors mention CHF, DM and malignancy in the text. Kindly address this discrepancy.

4. (Major concern) Results: Number of medications: It seems that the authors conducted logistic regression with either NLR>3.3 or NLR>4.5 as the dependent variable and number of medications as an independent, ***continuous ***variable. This is ***incorrect***. One cannot assume the number of medications to have a continuous effect. The authors need to change their analysis, and incorporate number of medications as a categorical variable with categories: zero (referent), 1, 2, 3 and 4 or more. There will be separate odds ratios for categories 1, 2, 3 and 4 or more (the authors may elect to choose different categories, but it is important that they use categories rather than a continuous variable). This may change the results, esp for NLR > 4.5.

5. (Major concern) Results: The authors need to explain in greater detail how they performed 'regression' analysis with NLR and ASA and NLR and RCRI. Was NLR treated as a continuous variable or in categories>3.3 or >4.5? What was the dependent variable? I am assuming that the authors performed logistic regression analysis with ASA or RCRI as the dependent variable and NLR as the independent variable in a continuous fashion, but this needs to be explicitly stated, since it helps in understanding what the odds ratios mean.

6. What about dividing NLR into tertiles or quartiles instead of using the said cut offs, since there are not established cut offs for NLR?

7. Discussion, first paragraph: The association that the authors observe in this study between NLR and CHF and NLR and malignancy is just that- an association. The authors cannot infer that this means that either NLR is associated with long term outcomes in CHF (ref 19) or prognostic value of NLR in cancer (ref 20). I would therefore strongly recommend the authors to remove those two lines from the first paragraph of discussion.

8. Discussion, Third paragraph (no. of meds): This may have to be re-written after the authors re-analyze the data with no. of meds as a categorical variable.

9. The authors do have interesting findings and I was hoping to see if preoperative NLR predicts outcomes (morbidity and mortality or major cardiac events) in the postoperative period. Moreover, if NLR does predict outcomes in the postoperative period, it may be used to re-classify the existing ASA and RCRI indices for non-cardiac surgeries. The authors should mention this in discussion or conclusion as future studies that may be undertaken in this subject.

I have read this submission. I believe that I have an appropriate level of expertise to confirm that it is of an acceptable scientific standard, however I have significant reservations, as outlined above.

# Peer review: Referee response for version 1

**DOI:** 10.5256/f1000research.6946.r8777

**Published:** 2015-05-28

**Authors:** Gareth L. Ackland, Mark Edwards

**Affiliations:** 1 Division of Medicine, University College London, London, UK; 2 University Hospital Southampton, University of Southampton, Southampton, UK

The authors have performed a cross sectional observational study examining the relationship between NLR, preoperative systemic illnesses and peri-operative risk markers in a large cohort of surgical patients. This makes a contribution to the existing literature by including patients from a variety of surgical specialties rather than focussing on specific surgical pathologies.

They found that increased NLR at two cut-offs was independently associated with pre-existing cardiac failure and malignancy (confirming similar findings in other studies) – but not ischaemic heart disease - in a multivariable model. Overall the design, methods and analysis are appropriate and the authors’ conclusions are balanced and justified.

The study authors may consider the following points which may strengthen this work:

1. The abstract and results text state that diabetes was one of the pre-existing co-morbidities associated with increased NLR in the multivariate model, however this is not consistent with the results tables.
2. The regression models examining ASA / RCRI and NLR could be described more clearly. Were they separate univariate models or part of the wider multivariate models including co-morbidities? If the latter, the extent of co-linearity between these risk scores and the presence of co-morbidities would be useful to know.
3. As the authors point out, this study has not included clinical outcomes and did not aim to examine NLR as a prognostic marker. With this in mind, could NLR have been examined as a continuous variable in the regression model rather than being dichotomised? Dichotomising continuous data may have led to a loss of statistical power ( Altman and Royston, 2006). Also, although the NLR cut-offs examined have been justified based on prior related literature, giving these cut-offs (3.3 and 4.5) might imply to the casual reader that these are prognostically important values, whereas this needs further exploration as the authors acknowledge.

We have read this submission. We believe that we have an appropriate level of expertise to confirm that it is of an acceptable scientific standard.
